# Diagnostic and Clinical Value of Specific Autoantibodies against Kelch-like 12 Peptide and Nuclear Envelope Proteins in Patients with Primary Biliary Cholangitis

**DOI:** 10.3390/biomedicines10040801

**Published:** 2022-03-29

**Authors:** Alicja Bauer, Andrzej Habior, Damian Gawel

**Affiliations:** 1 Department of Biochemistry and Molecular Biology, Centre of Postgraduate Medical Education, 01-813 Warsaw, Poland; damian.gawel@cmkp.edu.pl; 2 Clinic of Polish Gastroenterology Foundation, 02-653 Warsaw, Poland; ahab@coi.waw.pl; 3 Department of Cell Biology and Immunology, Centre of Postgraduate Medical Education, 01-813 Warsaw, Poland

**Keywords:** PBC, autoantibodies, glycoprotein gp210, nucleoporin p62, KLHL12 peptide

## Abstract

Primary biliary cholangitis (PBC) is a chronic autoimmune liver disease characterized by the presence of antimitochondrial and antinuclear antibodies in patients’ serum. Here, we analyzed the reactivity of autoantibodies against a novel autoantigen, kelch-like 12 (KLHL12) protein, in a cohort of 138 PBC and 90 non-PBC patients. Additionally, we compared the reactivity of KLHL12 with antinuclear envelope antibodies: anti-gp210, anti-p62, and anti-LBR. Commercially available kits and an ‘in-house’ ELISA were used in the studies. Antinuclear envelope antibodies were detected in 65% of PBC patients and the presence of these antibodies was observed more frequently in patients diagnosed with later stages (III/IV) of PBC, according to Ludwig’s classification (*p* < 0.05) and were found to correlate with a higher concentration of bilirubin. Overall, anti-KLHL12 antibodies were found more frequently in PBC patients than in non-PBC controls (*p* < 0.001). Anti-KLHL12 antibodies were detected in 36% of the tested PBC cohort, including PBC patients negative for antimitochondrial antibodies. Presence of anti-KLHL12 was also associated with a higher concentration of bilirubin and correlated with fibrosis (*p* < 0.05). Anti-KLHL12 antibodies were detected in 30% of PBC individuals positive for antinuclear envelope antibodies, while anti-KLHL12 and antinuclear envelope antibodies were found in 17% of all PBC cases. Concluding, our data confirm that antibodies against the KLHL12 protein are highly specific for PBC and when used in combination with other markers, may significantly increase the diagnosis of PBC.

## 1. Introduction

Primary biliary cholangitis (PBC) is a chronic, progressive, immune-mediated cholestatic liver disease with a strong genetic basis [1,2,3,4,5,6,7,8,9,10]. The most characteristic immunological features of this entity are anti-mitochondrial antibodies [11,12,13,14]. The M2 fraction of antimitochondrial antibodies (AMA M2), directed against the 2-oxoacid-dehydrogenase complex of the inner mitochondrial membrane, is detected in up to 95% of PBC patients. In addition, different types of antinuclear antibodies (ANAs) can be found in approximately 50% of PBC patients [15,16,17,18,19,20]. There are several nuclear structures recognized as targets for ANAs in PBC [21,22,23,24,25,26,27,28,29]. Some ANAs specifically target nuclear envelope (NE) proteins [30,31,32,33,34,35]. ANAs directed against NE proteins, such as anti-gp210 antibodies, are not common; nevertheless, they seem to be highly specific for PBC [36,37]. Some data indicate that anti-gp210 antibodies may be used as a marker for unfavorable prognosis of PBC [38,39,40]. Nucleoporin p62 is another protein associated with the NE. Wesierska-Gadek et al. (2007; 2008) found anti-p62 antibodies in about 50% of PBC patients [41,42], while Miyachi et al. (2003) showed their presence in 13% of PBC cases [43].

Antibodies against the kelch-like 12 (KLHL12) peptide were recently identified as a new biomarker for PBC and notably indicate patients who are negative for conventional autoantibodies [44]. Nevertheless, the prevalence of anti-KLHL12 antibodies in different geographical areas has rarely been reported [45]. The kelch-like family of proteins, consisting of 66 KLHL genes, appears to be involved in multiple cellular functions including cell structure, cellular communication, transcriptional regulation, collagen export, and ubiquitination of proteins through interactions with the cullin-ring E3-ligases [46,47]. KLHL12, which is located inside the nucleus, is part of this evolutionarily conserved superfamily and is crucial for collagen export [46,48]. The KLHL12 antigen was detected using microarray, proteomic, and modified enzyme-linked immunosorbent assay (ELISA) analyses [49]. These new antibodies have not been widely employed in practice and only a few reports can be found [44,45,49].

In the present study, we determined specific antibodies directed against the KLHL12 peptide in sera of Polish PBC patients and compared them to autoantibodies against anti-NE proteins, including anti-gp210, anti-p62, and anti-LBR. Additionally, we compared the presence and level of all these antibodies to biochemical and histological parameters, and evaluated their significance for the diagnosis of PBC.

## 2. Materials and Methods

### 2.1. Patients

Serum samples were collected from 138 patients (131 women, 7 men; median age: 50 age range: 26–70 years), diagnosed at the Centre of Postgraduate Medical Education (Warsaw, Poland). The diagnosis of PBC was established using generally accepted criteria according to the practical guidelines of the European Association for the Study of the Liver(EASL) for PBC [50,51]. A liver biopsy was performed in all cases. Patients positive for the hepatitis B surface antigen (HBsAg), anti-hepatitis A virus IgM, hepatitis C virus, and patients with alcoholism and autoimmune hepatitis (AIH)/PBC overlap syndrome were excluded from the study. In most patients, the diagnosis was made within one year after the onset of symptoms. The main criteria for outcome measure were time of death from liver failure or time to liver transplantation. For the analysis, we selected patients who had no other comorbidities detected at the time of the study. None of the patients had previous gastrointestinal disease. However, shortly after the serum was collected, 6 patients were diagnosed with other autoimmune diseases: 3 with Sjogren’s syndrome; 1 with rheumatoid arthritis; 1 with Hashimoto’s disease; 1 with systemic lupus erythematosus. The tested antibodies were not detected in any of these patients.

The control group consisted of 40 patients (16 females, 24 males; median age: 47; age range: 23–67 years) with primary sclerosing cholangitis (PSC) and 20 patients with AIH (15 females, 5 men; median age: 47 years; age range: 19–67 years). Additionally, serum samples from 30 healthy adult blood donors (22 females, 8 men; median age: 33 years; age range: 20–52 years) were collected at the Warsaw Blood Bank. The study protocol was conducted in accordance with the ethical guidelines of the Declaration of Helsinki and was approved by the Ethical Committee of the Centre of Postgraduate Medical Education, Warsaw, Poland (approval number 71/PB/2019). Written informed consent was obtained from all patients.

### 2.2. Detection of Antibodies

#### 2.2.1. Detection of Anti-gp210 Antibodies and AMA M2

Anti-gp210 antibodies and AMA M2 were determined using commercially available ELISA kits (QUANTA Lite^®^ gp210; Inova Diagnostics, USA and QUANTA Lite^®^ M2 EP-MIT3, respectively; Inova Diagnostics, San Diego, CA, USA), according to the manufacturer’s instructions. Intra-assay performance of these kits was 4.6% and 2.9%, respectively, while their inter-assay performance was 5.8% and 6.1%, respectively.

#### 2.2.2. Detection of Anti-Nucleoporin p62 Antibodies

The level of anti-nucleoporin p62 (anti-p62) was evaluated by an ‘in-house’ ELISA, as previously described [52]. The antibody levels were calculated with reference to standard serum. Results > 20 units/mL were considered positive. The intra-assay performance of the used ‘in-house’ ELISA test was on average 4.5%, while the inter-assay coefficient of variation was equal to 11%.

#### 2.2.3. Detection of Anti-LBR Antibodies

Anti-lamin B receptor (anti-LBR) antibodies were determined using an ‘in-house’ ELISA. Wells of flat-bottom microtiter plates (Costar, Corning, NY, USA) were coated with the LBR recombinant protein (Abcam, Cambridge, UK) dissolved in bicarbonate buffer (pH 9.9), then saturated with 1% bovine serum albumin (BSA; Sigma-Aldrich, Steinheim, Germany) in phosphate buffered saline (PBS; pH 7.4; HyClone; Cytiva, Marlborough, MA, USA), and washed with PBS supplemented with 0.1% Tween (PBST; Sigma-Aldrich). Next, the tested sera (diluted 1:100 in PBS) were incubated in coated plates for 1 h at room temperature (RT) with horseradish peroxidase-conjugated antibodies to human IgG (dilution 1:50,000; Daco A/S; Glostrup, Denmark) in PBST. The color reaction was developed by adding 0.1 mL of tetramethylbenzidine (TMB; SERVA Electrophoresis GmbH, Heidelberg, Germany) and stopped using 0.5 M H_2_SO_4_. The optical density (OD) was measured at 450 nm with an automatic plate reader (Multiscan RC, Labsystem; Vantaa, Finland). The final levels of antibodies were calculated with reference to standard serum, which had been diluted to five different concentrations (10, 30, 50, 200, and 500 units/mL). Results lower than 15 units/mL were arbitrarily determined as negative. The intra-assay performance of our ELISA ‘in-house’ test was on average 4.8% and the inter-assay coefficient of variation was equaled to 10.5%.

#### 2.2.4. Detection of Anti-KLHL12 Antibodies

Anti-KLHL12 antibodies were detected using an ELISA test developed at the Centre of Postgraduate Medical Education in Poland, using the recombinant KLHL12 protein (Abnova, Taipei, Taiwan). Flat-bottom microtiter plates (Costar, Corning, NY, USA) were coated with a solution of the KLHL12 recombinant protein in bicarbonate buffer (pH 9.9), then saturated with 1% BSA in PBS, and washed with PBST. The tested sera (1:100 in PBS) were incubated on coated plates for 1 h at RT with horseradish peroxidase-conjugated human IgG antibodies (Daco A/S, dilution 1:50,000 in PBST). The color reaction was developed by adding 0.1 mL of TMB (SERVA Electrophoresis GmbH) and stopped using 0.5 M H_2_SO_4_. The optical density (OD) was measured at 450 nm with an automatic plate reader (Multiscan RC, Labsystem, Vantaa, Finland). The levels of antibodies were calculated with reference to our standard serum diluted to: 10, 20, 50, 200, and 400 units/mL. Results lower than 30 units/mL were arbitrarily determined as negative. The intra-assay performance of the developed ‘in-house’ ELISA test was on average 4.3%. The calculated inter-assay coefficient of variation was equaled to 10.3%.

### 2.3. Statistical Analysis

Prevalence rates were compared between groups using the chi-squared test and Fisher’s exact test. Continuous data were summarized as mean ± SD (standard deviation), and categorical data were summarized as frequencies. Continuous variables were evaluated using the Mann–Whitney test and were expressed as median ± interquartile range (IQR). A *p*-value below 0.05 was considered statistically significant. All statistical analyses were performed using the Statistica 8.0 software (Stat-Soft; Cracow, Poland) and MedCal for Windows, version 7.4.1.0 (MedCal Software; Mariakerke, Belgium). Statistical analysis of the ROC curve was performed using Prism software (GraphPad; La Jolla, CA, USA) and MedCal version 7.4.1.0 (MedCal Software).

## 3. Results

### 3.1. Clinical, Histological, and Laboratory Features of PBC Patients and Control Groups

The clinical, histological, and laboratory characteristics of PBC patients are presented in Table 1.

It was determined that the total bilirubin levels were higher in over 50% of the tested samples. AMA M2 was detected in 82% of patients’ sera. Activity of AST and ALT was elevated in 73% and 62% of sera from PBC patients, respectively. Over 70% of patients presented increased activity of AP and γ-GT, while a decreased level of albumin was observed in 40% of PBC patients.

### 3.2. Occurrence and Diagnostic Value of Anti-Nuclear Envelope Antibodies

In the tested PBC patients, anti-NE antibodies were found in 76 out of 138 samples (55%). Anti-gp210 and anti-p62 antibodies were detected in 65 (47%) and in 39 (28%) out of 138 PBC patients, respectively. Among PBC patients, anti-LBR antibodies were found with a frequency of 15% (21/138), while no anti-LBR antibodies were detected in the pathological controls. None of the examined antibodies were found in any of the healthy controls. In the control group, among PSC patients, anti-gp210 and anti-p62 antibodies were found in one patient (2.5%), respectively. Among AIH patients, we also determined anti-p62 antibodies in only one sample (5%). The summary of sensitivities, specificities, and positive and negative predictive values for each detected antibody in patients with PBC is presented in Table 2.

We also checked the occurrence of the tested antibodies in the studied group of females only, as well as in the group of males. In the tested PBC female patients, anti-NE antibodies were found in 71 out of 131 samples (54%) and in the male group, anti-NE antibodies were found in 5 out of 7 samples (71%). This difference was not statistically significant. The data obtained for the female group did not differ from the data obtained for the entire PBC cohort.

Specificity of anti-NE proteins for diagnosis of PBC in the whole group of patients was 95.4%. The difference between proportions of anti-NE-positive patients and controls was 0.560 (95% C.I., 0.438–0.665, *p* < 0.0001). In our study, the positive predictive value (PPV) of anti-gp210 and/or anti-p62 was 89.2%, and the negative predictive value (NPV) was 76.4%. The accuracy of these tests and their ability to differentiate PBC patients and healthy cases was 80.3%.

**Figure 1:**
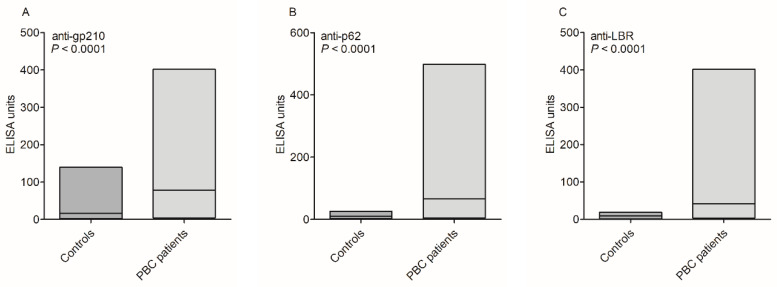
Levels of anti-nuclear envelope antibodies in sera of patients with PBC and the control group: (**A**) anti-gp210, (**B**) anti-p62, (**C**) anti-LBR.

The levels of anti-gp210, anti-p62, and anti-LBR antibodies in sera of PBC patients and control groups are presented in Figure 1.

There was a significant difference between PBC patients and the control group: 148 U/mL vs. 45 U/mL, *p* < 0.0001 for anti-gp120 antibodies, and 211 U/mL vs. 16 U/mL, *p* < 0.0001 for anti-p62 antibodies and 176 U/mL vs. 10 U/mL, *p* < 0.0001 for anti-LBR antibodies. Nearly 50% of patients showed a high level (above 150 U/mL) of anti-gp210, anti-p62, or anti-LBR antibodies.

**Figure 2:**
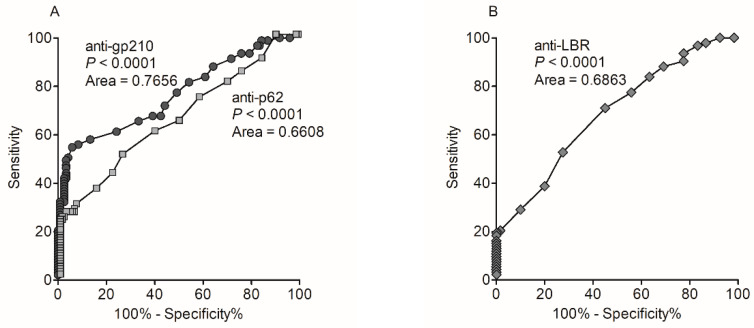
Receiver operating characteristic (ROC) curve analysis for serological detection of PBC anti-gp210 and anti-p62, and anti-LBR antibodies in PBC: (**A**) anti-gp210 and anti-p62, (**B**) anti-LBR.

Receiver operating characteristic (ROC) curve analysis for serological detection of anti-gp210 anti-p62 and anti-LBR in PBC samples is shown in Figure 2.

The determined area under the ROC curve (AUC) was greatest for anti-gp210 antibodies (0.7656). The values of AUC calculated for anti-LBR and anti-p62 autoantibodies were 0.6863 and 0.6608, respectively.

**Figure 3:**
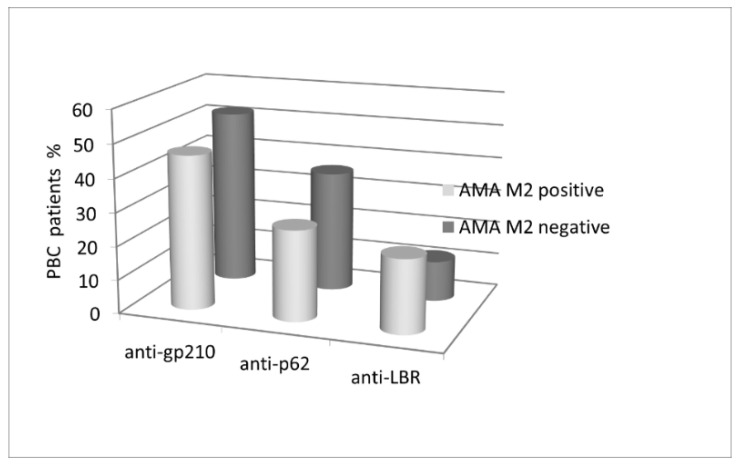
Prevalence of antinuclear antibodies in the studied AMA M2-positive and AMA M2-negative populations.

We also attempted to correlate anti-gp210, anti-p62, and anti-LBR serum antibodies with AMA M2 (Figure 3).

Anti-gp120 were detected in 52% of AMA M2-negative vs. 46% of AMA M2-positive patients. Similarly, anti-p62 were observed more frequently in AMA M2-negative (36%) than in AMA M2-positive (27%) patients. In contrast, lower levels of anti-LBR were observed in AMA M2-negative (12%) than in AMA M2-positive (22%) samples. Nevertheless, these differences were not statistically significant (*p* > 0.05).

Among the 25 AMA M2-negative patients, two (8%) were positive for all three tested antibodies (anti-gp210, anti-p62, anti-LBR). Only four AMA M2-negative patients (16%) were positive for at least two activities. Among the 113 AMA M2-positive patients, eight (6%) presented reactivity to all three tested antibodies, while 22 patients (19%) were positive for at least two activities. In the AMA M2-negative group of PBC patients, the positive detection rate for the combined detection of anti-gp210, anti-p62, and anti-LBR antibodies was 76% (19/25) and the accuracy reached 90%.

Among the 24 AMA M2-negative female patients, 18 (75%) were positive for anti-NE antibodies.

### 3.3. Occurrence and Diagnostic Value of Anti-KLHL12 Antibodies

Anti-KLHL12 antibodies were detected more frequently in PBC compared to non-PBC controls (*p* < 0.001; Table 3).

In the tested PBC female patients, anti-KLHL12 antibodies were found in 47 out of 131 samples (36%), while in the male group, anti-KLHL12 antibodies were found in two out of seven samples (29%).

**Figure 4:**
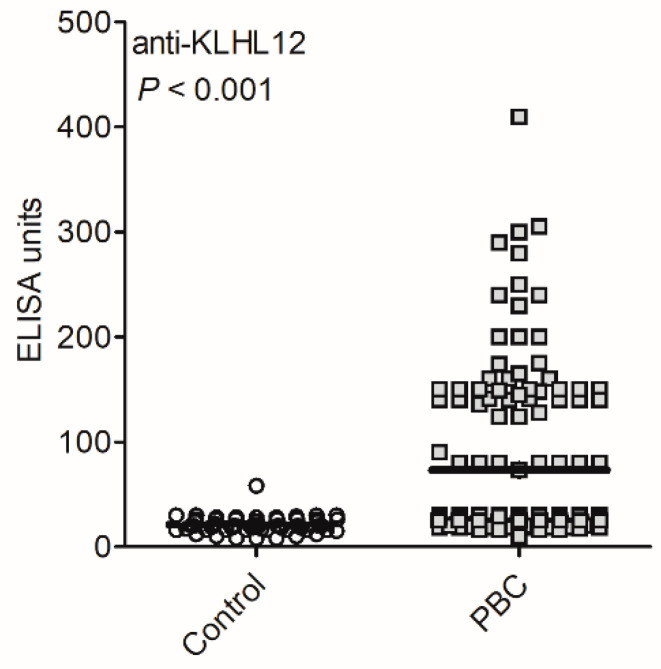
Anti-KLHL12 autoantibodies in sera of PBC patients.

Specificity of anti-KLHL12 antibodies for diagnosis of PBC in the group of patients was 97%. The positive predictive value (PPV) of anti-KLHL12 antibodies in the diagnosis of PBC was 94% and the negative predictive value (NPV) was 49%. The positive and negative likelihood ratios determined from suitable sensitivities and specificities were 10.4 and 0.7, respectively. The levels of anti-KLHL12 autoantibodies in sera of PBC patients and control groups are shown in Figure 4.

The mean level of antibodies in the group of PBC patients was significantly higher than in the control group: 73 U/mL vs. 21 U/mL, *p* < 0.001. Over 30% of anti-KLHL12-positive PBC patients demonstrated enhanced levels of antibodies (>100 U/mL).

**Figure 5:**
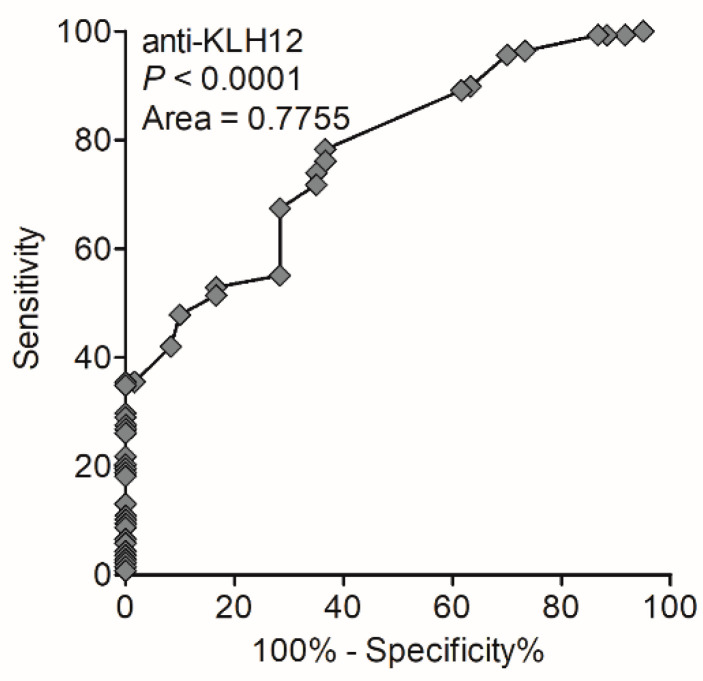
Receiver operating characteristic curve analysis for serological detection of anti-KLHL12 autoantibodies in PBC patients.

We generated a ROC curve. The sensitivity and specificity were calculated for the cut-off value of 30 arbitral units (Figure 5).

The analysis showed that the combined positive detection rate of anti-NE and anti-KLHL12 antibodies in PBC was 60%, while the accuracy of the tests and their ability to differentiate PBC patients and healthy cases was found to be 76%.

**Figure 6:**
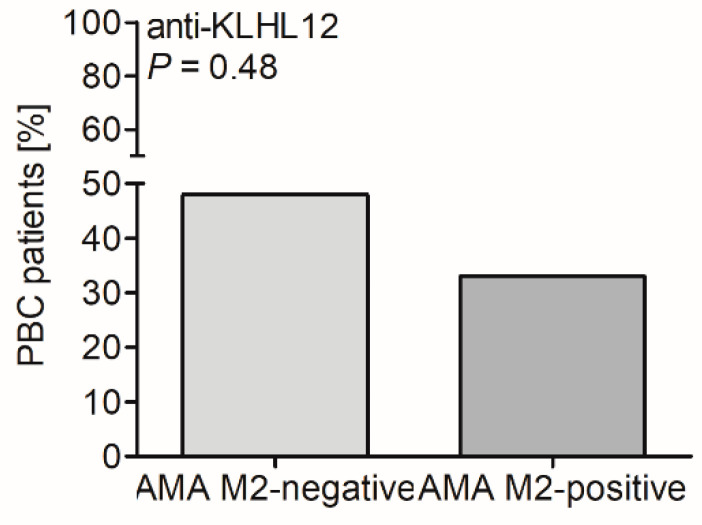
Prevalence of anti-KLHL12 antibodies in the AMA M2-positive and AMA M2-negative PBC cohorts.

We also compared the presence and level of serum anti-KLHL12 antibodies with the presence of AMA M2 in PBC patients. Anti-KLHL12 antibodies were identified in 38% of AMA M2-negative vs. 30% of AMA M2-positive samples (Figure 6).

These differences were not statistically significant, but anti-KLHL12 antibodies were more frequently detected in the AMA M2-negative group of PBC patients (*p* = 0.48).

The mean levels of the anti-KLHL12 antibodies in the AMA M2-positive and AMA M2-negative groups differed significantly (71.0 ± 57.4 vs. 48.1 ± 42.5; *p* = 0.0283). Most importantly, anti-KLHL12 antibodies were present in 38% of AMA M2-negative PBC patients. Addition of this biomarker to conventional PBC assays improves the serological sensitivity of the AMA M2-negative group of PBC subjects (from 48.3% to 68.5%).

We also studied the prevalence of anti-KLHL12 antibodies in the anti-NE-positive and anti-NE-negative PBC populations. Anti-KLHL12 antibodies were identified in 39% of the anti-NE-negative vs. 33% in the anti-NE-positive PBC samples.

We observed an autoimmune reaction against multiple nuclear components in the evaluated subgroups of PBC patients. Two (8%) out of the tested 25 AMA M2-negative patients and six (5%) out of 113 AMA M2-positive patients were positive for all four reactivities (anti-gp210; anti-p62; anti-LBR; anti-KLHL12). Interestingly, in six (4%) patients only anti-KLHL12 antibodies were found.

We evaluated the diagnosability by combining five markers: anti-gp210, anti-p62, anti-LBR, anti-KLHL12, and AMA. The test’s sensitivity increased significantly from 82% to 93% for detection of AMA only (*p* = 0.0093) and a slight improvement in accuracy was also observed (from 89% to 94%).

### 3.4. Biochemical Features of PBC Patients According to the Status of Anti-NE and Anti-KLHL12 Antibodies

Comparison of groups of PBC patients who were positive and negative for anti-NE and/or anti-KLHL12 antibodies showed that the symptoms of the disease began at the same age in each group of patients. The results of laboratory tests performed at the time of diagnosis were comparable in patients positive and negative for both analyzed autoantibodies, with the exception of bilirubin levels. A correlation between the presence of these autoantibodies and a higher concentration of bilirubin was found. Patients with positive reactivity for anti-NE antibodies (positive for at least one of three anti-NE reactivities: anti-gp210, anti-p62, anti-LBR) and anti-KLHL12 antibodies had higher levels of total bilirubin (2.4 vs. 1.7, *p* = 0.016 and 2.6 vs. 1.5, *p* = 0.037, respectively). Data from biochemical analyses performed at the time of diagnosis in 138 PBC patients, according to the anti-NE and anti-KLHL12 antibodies status, are presented in Table 4.

**Figure 7:**
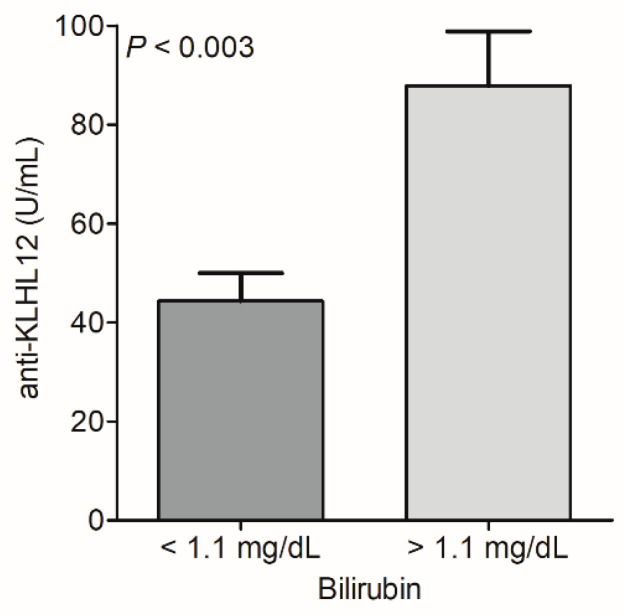
Level of anti-KLHL12 antibodies and bilirubin concentration in serum of PBC patients.

The bilirubin levels were significantly higher in the group of KLHL12-positive patients (*p* < 0.05). In patients with elevated levels of bilirubin, the mean level of antibodies was ~90 U/mL, while in patients with a normal bilirubin concentration, a level of ~50 U/mL was observed (Figure 7).

### 3.5. Autoantibodies Directed against Nuclear Envelope Proteins and KLHL12 Antibodies, and the Survival of Patients

**Figure 8:**
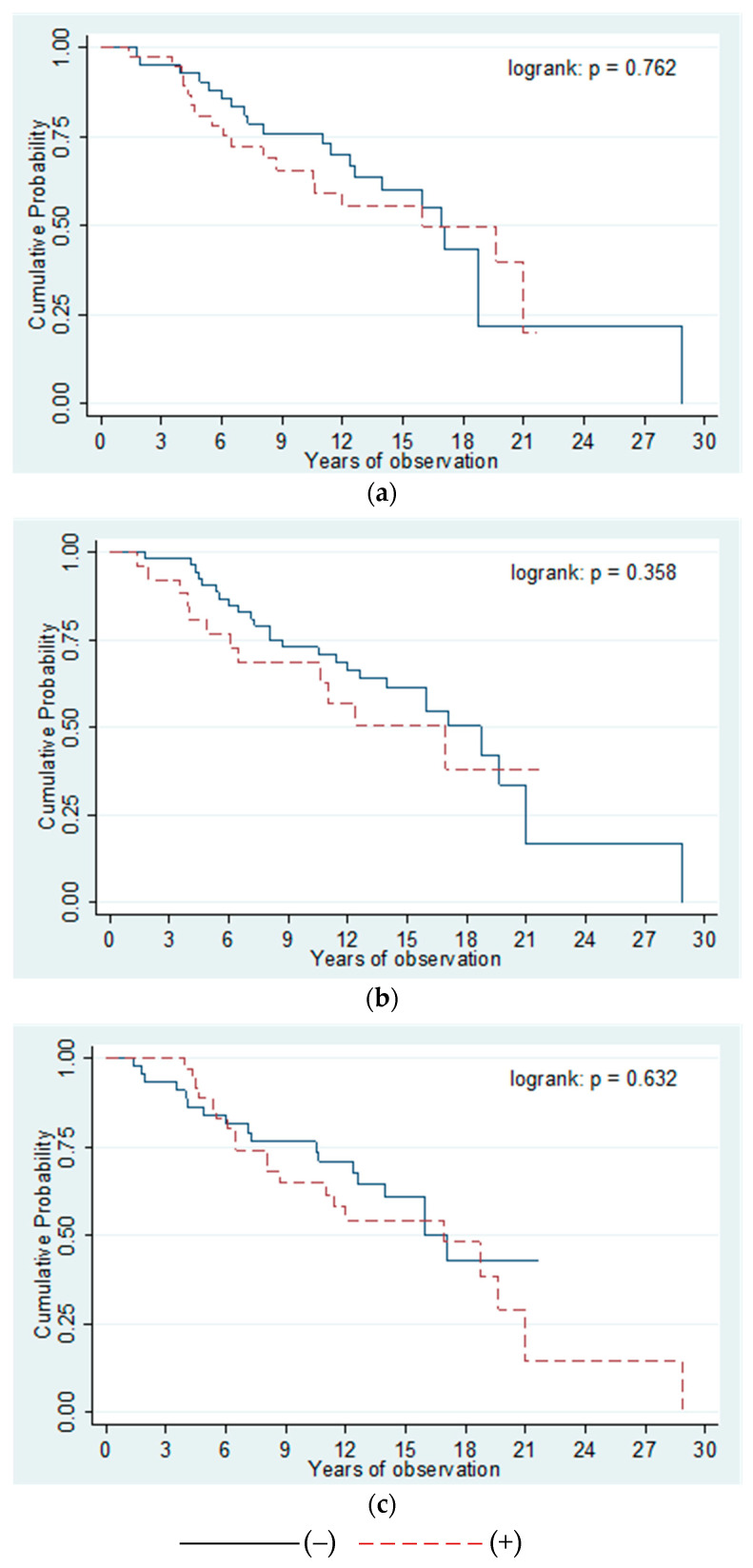
Kaplan–Meier curves demonstrating the survival of patients positive and negative for anti-nuclear envelope antibodies. (**a**) Anti-gp210 antibodies; (**b**) Anti-p62 antibodies; (**c**) Anti-LBR antibodies.

Analysis of the survival rate performed in patients positive and negative for anti-gp210, anti-p62, and anti-LBR (Figure 8) demonstrated that these autoantibodies did not affect the length of life or time to liver transplant in PBC patients.

There was no direct association between anti-gp210, anti-p62, and anti-LBR antibodies, and the early onset or significantly shorter survival of patients. Nevertheless, in the group of patients characterized by presence of at least two types of the tested antibodies, the patient’s survival time or time to liver transplant were more than 4-times shorter than in the group without anti-gp210, anti-p62, or anti-LBR antibodies (OR = 4.375; *p* = 0.0432).

Moreover, in this small group of patients characterized by presence of all three types of these antibodies, we found over 5-times more deaths or transplants than in the group without antibodies (OR = 5.200; *p* = 0.036).

**Figure 9:**
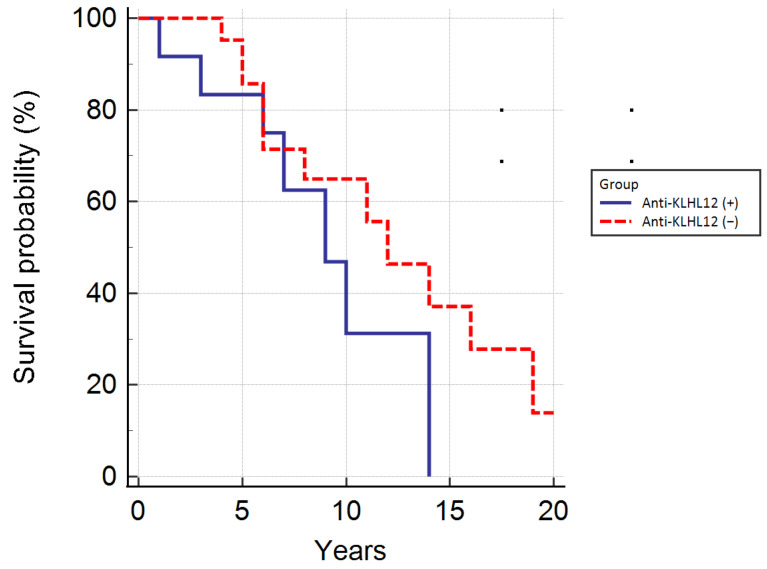
Kaplan–Meier curves demonstrating the survival of patients positive and negative for the anti-KLHL12 antibodies.

Analysis of the survival of patients positive and negative for the anti-KLHL12 antibodies (Figure 9) showed that presence of these autoantibodies also does not correlate with the length of life or time to liver transplant in PBC patients (*p* = 0.07).

Although the difference was not statistically significant, the survival time of patients or the period to liver transplant was shortest for people with these antibodies, in comparison to anti-NE antibodies.

### 3.6. Analysis of the Correlation between Histological Parameters of PBC Patients, and Autoantibodies Directed against Nuclear Envelope Proteins and the KLHL12 Protein

**Figure 10:**
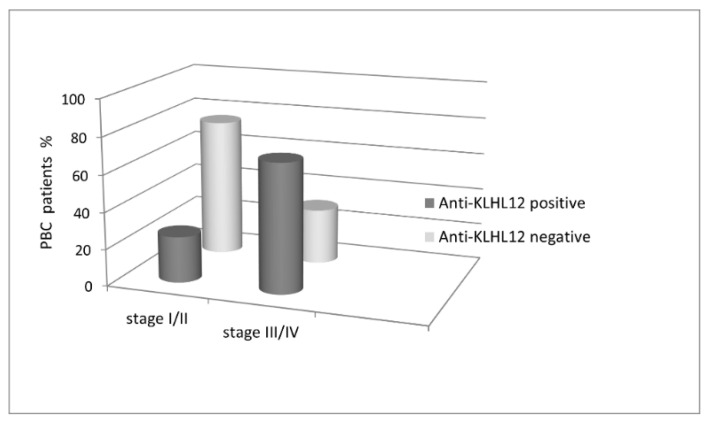
Prevalence of antinuclear envelope antibodies in the studied population according to stage of Ludwig’s classification.

We assessed histological material collected from PBC patients and classified it into two groups based on presence or absence of anti-NE antibodies. Among 76 anti-gp20- and/or anti-p62- and/or anti-LBR-positive PBC patients, 21% were classified into stages I/II, and 79% into stages III/IV, according to Ludwig’s classification (Figure 10).

**Figure 11:**
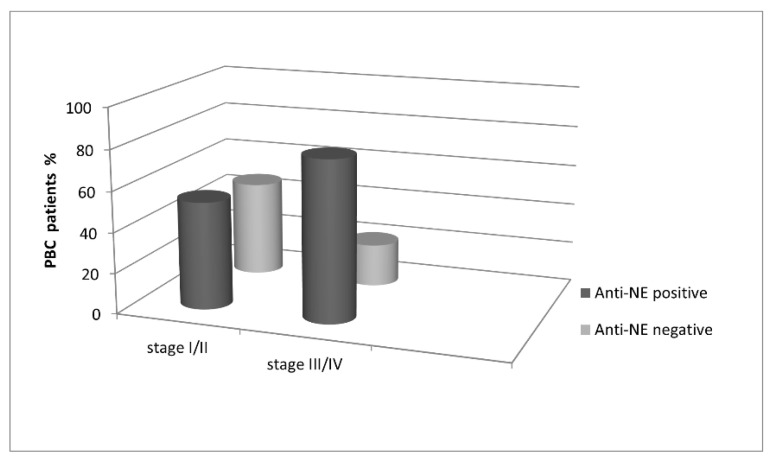
Presence of anti-KLHL12 antibodies and the stage of liver fibrosis in PBC patients.

Among 82 PBC patients with early histological stages (I/II) of the disease, 43 (52%) were anti-NE-positive. In the group of PBC patients with advanced histological stages (III/IV), 41 out of 52 (79%) were anti-NE-positive (*p* = 0.002). Statistically, more patients with stages III/IV in the anti-gp210-positive than anti-gp210-negative subgroups (43% vs. 14%, *p* < 0.001) were found. The same pattern was observed for patients with anti-p62 antibodies (42% vs. 21%, *p* = 0.041 and 16% vs. 4%, *p* = 0.048) and anti-LBR antibodies (67% vs. 31%; *p* = 0.002). Presence of the anti-KLHL12 antibodies in sera of PBC patients also correlated with the stage of liver fibrosis (Figure 11).

Among 82 PBC patients with early histological stages (I/II) of the disease, only 20 (24%) were positive for anti-KLHL12 antibodies. We found a statistically significant difference in comparison to the group of PBC patients with advanced histological stages (III/IV), where 39 out of 52 (75%) subjects were positive for anti-KLHL12 antibodies (*p* < 0.0001).

## 4. Discussion

PBC is primarily characterized by presence of AMAs, with the most important antigens being PDC-E2, OGDC-E2, and BCOADC-E2 [11,12,13]. As non-invasive tests for identification of autoantibodies as disease markers are useful for diagnosis of patients, we analyzed the immune response against gp210, p62, and LBR antigens, and the KLHL12 protein in PBC. Anti-NE antibodies have been considered as a pathognomonic element of PBC [36], however, a significant variation in their prevalence (between 10% and 50%) has been reported. Antibodies against integral glycoproteins of the nuclear pore membrane, gp210 and p62, have been reported [16,17,18], and are associated with pathogenesis, progression, and severity of PBC [31,32,33]. Studies performed in Western Europe, North America, and East Asia have shown high levels of anti-gp210-specific antibodies in PBC patients [17,18,19,53,54], which has also been confirmed in the Polish population. The measured sensitivity of anti-gp210 antibodies in PBC was found to be 44% [32], which is higher than that reported from other parts of the world: Japan–26% [43], Italy–18% or 27% [30,35,55], and Spain–33% [56]. Huang et al. (2019) proposed usage of the gp210 antibody for early diagnosis of PBC [31]. In our study, antibodies directed against the p62 protein were found in 28% of the screened sera of the tested PBC patients, which was similar to the frequency reported earlier by Wesierska-Gadek et al. (2008) [41]. However, the specificity of anti-NE antibodies for PBC was greater than 97%, which seems to be remarkably high. A slightly higher prevalence of anti-gp210 and anti-p62 antibodies in AMA M2-negative PBC patients was observed. Even though this difference was not statistically significant, cases with advanced histological stages were much more common among anti-NE-positive and AMA M2-negative patients. Previous reports have highlighted the correlation between anti-gp210 and anti-p62 antibodies, and the clinical outcome of PBC. Nakamura and co-workers (2005) analyzed clinical, immunological, and histological data on 71 Japanese PBC patients in relation to the presence of the anti-gp210 antibody. They suggested that this autoantibody is a promising prognostic marker of a poor outcome of the disease [57]. Invernizzi et al. (2001) demonstrated a strong association between presence of autoantibodies against nuclear pore complexes with more active and severe cases of PBC [55]. Bogdanos et al. (2007) suggested a more progressive form of the disease in patients with ANAs [58]. Haldar et al. (2021) confirmed that presence of anti-gp210 is associated with an adverse phenotype, lack of response to treatment, and reduced transplant-free survival in the cohort of PBC patients [59]. The regional differences in the prevalence of anti-nuclear antibodies were explained by environmental and genetic factors, rather than technical differences in the determination of anti-gp210 [56]. Yang and co-workers (2004) studied presence of anti-nuclear antibodies in a large Toronto and Mayo Clinic cohort of PBC patients using immunofluorscence and found that these antibodies are associated with earlier development of liver failure [60]. They found that Wesierska-Gadek et al. (2008) reported the presence of an antibody against the nuclear pore complex in patients likely to experience an unfavorable clinical course and more rapid progression of the disease [37]. Contrary to these results, we found no direct association between presence of anti-nuclear antibodies and poor prognosis of PBC. These data stay in accordance with some of the previously published findings [43,58].

Our data also revealed that the serum bilirubin concentration at the time of diagnosis was an independent and the only risk factor for poor prognosis of PBC. Excluding bilirubin, our subjects with or without anti-gp210 or anti-p62 antibodies presented similar biochemical characteristics. Neither anti-gp210 nor anti-p62 were distinct independent risk factors for rapid progression and liver failure in PBC patients. Nevertheless, PBC patients positive for these antibodies were frequently classified into later stages (III/IV), according to Ludwig’s classification. Sfakianaki et al. (2010) obtained similar results, but contrary to us, they also found a correlation between positive anti-gp210 antibodies and a short survival period of patients [61]. Therefore, it seems that presence of anti-p62 antibodies or simultaneous occurrence of anti-p62 and anti-gp210 antibodies may be of much greater significance for the prediction of a worse course of the disease. LBR autoantibodies are only rarely detected in patients with PBC (2% to 10%) [62], hence they are not of general diagnostic utility. Their prognostic significance is also unknown. Due to the low sensitivity of these antibodies in PBC, they have also not been extensively studied. We decided to analyze our relatively small group of anti-LBR positive patients in more detail. We observed that autoantibodies directed against LBR were highly specific for PBC, but were present in only 15% of our PBC patients, including AMA M2-negative subjects. In contrast, anti-LBR antibodies were not found in the pathologic and healthy controls. The detected sensitivity was higher than the values previously reported [63]. The high specificity and PPV for PBC is very interesting. Presence of anti-LBR does not correlate with patients’ survival rate, but is connected with liver fibrosis.

Anti-KLHL12 antibodies have not been widely employed in practice and only a few reports can be found. The prevalence of these antibodies in different geographic areas has also not been reported and only one study demonstrated results from five different sites examined from North America and Europe [45]. Norman et al. (2015) have shown that anti-KLHL12 antibodies are present in both AMA M2-positive, and importantly, AMA M2-negative patients [40,46]. In a cohort of 366 patients with PBC, ~40% of the 277 AMA M2-positive patients were positive for anti-KLHL12 antibodies, while 53 out of 89 AMA M2-negative patients were positive for anti-KLHL12 antibodies. The specificities of antibodies were 96–97%. The Norman et al. international multi-center study included a pilot trial of 40 patients from Poland in which a commercially non-available INOVA kit was used [45]. In contrast, in our study, we analyzed sera from a larger group of Polish patients and we also developed an ‘in-house’ ELISA, in which a recombinant protein, KLHL12, was used to detect autoantibodies directed against KLHL12 in PBC patients and controls. We verified the seropositivity of KLHL12 in the PBC group of Polish patients and found that the frequency of these antibodies in PBC was significantly higher compared to the control groups. These anti-KLHL12 antibodies detected using the ELISA ‘in-house’ method presented very high specificity. In our study, the positive rate of the anti-KLHL12 antibodies was 36%, which was slightly higher than in the rest of Europe and the United States (22%, 33.3%, respectively) [45]. Norman et al. also reported that KLHL12 antibodies have higher sensitivity than anti-gp210 antibodies [44]. We have not confirmed this observation in our group of patients. A coexistence of different antibodies was observed, which suggests an autoimmune reaction against multiple nuclear components in some PBC patients. A few patients only had anti-KLHL12 antibodies. We compared the prevalence of anti-KLHL12 antibodies in the AMA M2-positive and AMA M2-negative PBC populations and no statistically significant difference was observed. The level of anti-KLHL12 antibodies was slightly enhanced in the group of AMA-positive patients.

We analyzed the immune response against the KLHL12 protein in PBC patients and linked the obtained data with biochemical and histological parameters. Interestingly, we found an association between the presence of these autoantibodies and a higher concentration of bilirubin. For the diagnosis of PBC, specificity of the disease marker is one of the most important criteria. The bilirubin levels in sera of patients positive for anti-KLHL12 antibodies were higher than those in the anti-KLH12 antibodies negative group. The level of anti-KLHL12 antibodies in sera of PBC patients was also found to correlate with the stage of liver fibrosis. Combining anti-KLHL12 antibodies with available markers (MIT3, gp210, and sp100) increased the diagnostic sensitivity for PBC. Due to the anti-NE combination and the detection of anti-KLHL12 antibodies, the diagnostic sensitivity in PBC, especially in AMA-negative PBC, can be significantly improved from 48.3% to 68.5% in ELISA. That can reduce the risk of liver biopsy. The addition of tests highly specific for anti-KLHL12 antibodies to AMA and ANA serological assays significantly improves the efficacy of clinical detection and diagnosis of PBC, especially for AMA M2-negative subjects. To become globally adopted, it is important to validate these new biomarkers in different geographical areas. It is still unclear why anti-KLHL12 antibodies are present in patients with PBC and this will require further research.

In patients with chronic intrahepatic cholestasis, determination of serum AMA and PBC-specific ANA antibodies (immunofluorescence and/or specific anti-sp100/anti-gp210 testing by Western blotting or ELISA) is recommended as the next diagnostic step [51]. AMA positivity is found in more than 90% of patients with PBC, immunofluorescence 1/40, or immunoenzymatic reactivity observed during cholestatic serum liver testing, is highly specific to the disease [64]. The EASL recommends that in patients with cholestasis and no likelihood of systemic disease, a diagnosis of PBC can be based on elevated ALP levels and presence of AMAs at a titer of 1:40 [51]. AMA reactivity is only sufficient for diagnosis of PBC when combined with abnormal serum liver tests. EASL recommends that, in the correct context, a diagnosis of AMA-negative PBC can be made in patients with cholestasis and ANA-specific immuno-fluorescence (nuclear dots or perinuclear rims) or ELISA (using sp100 or gp210 antibodies) [51,65]. In contrast to anti-gp210 antibodies, the importance of antibodies against p62 in PBC is less recognized. Although the presence of anti-p62 is fairly rare, a positive ELISA result strongly supports the diagnosis of PBC. The high specificity of anti-p62 suggests that it may be considered as a significant serological marker of PBC, even when AMA, anti-gp210, and anti-sp100 antibodies are not detectable. Our study conducted on the group of PBC patients stays in accordance with other data which confirm very high specificity of anti-p62 [33,52]. The absence of classic PBC markers, such as AMAs and anti-gp210 or anti-sp100, can lead to a delay in diagnosis and treatment. Therefore, anti-p62 detection could be of crucial importance in PBC diagnosis. The explanation as to why antibodies to KLHL12 are higher in patients with PBC remains an interesting topic, although it is still unclear. Our study, as well as Norman et al.’s results [44,45], have demonstrated that anti-KLHL12 antibodies are novel, highly specific markers of PBC and, most importantly, they have been suggested to be promising new candidates in the clinical diagnosis of PBC. The addition of tests for highly specific anti-KLHL12 antibodies to AMA and ANA serological analyses considerably improves the effectiveness of clinical detection and diagnosis of PBC [44,45,49]. The level of anti-KLHL12 antibodies in sera of PBC patients is associated with the stage of liver fibrosis, which may be important in recognition of patients at risk of advanced disease or faster disease progression. However, it must be considered that immune markers should always be interpreted together with clinical findings by an experienced practitioner to avoid misdiagnosis.

## 5. Conclusions

Our data confirmed that there is a correlation between the presence of anti-KLHL12 antibodies, anti-nuclear envelope antibodies, liver fibrosis, and higher bilirubin concentrations in Polish PBC patients. Antinuclear antibodies of different specificity support the autoimmunity of PBC. The ability to detect them expands the diagnostic armamentarium of PBC-specific markers, especially in cases in which AMA are not detectable, in asymptomatic patients, and for early diagnosis of PBC. High specificity of these antibodies can imply that they may take part in the pathogenesis of the disease.

It appears that KLHL12 antibodies present predictive significance for more rapid PBC progression and can be considered as a risk factor for poor prognosis. As we confirmed that these antibodies are highly specific for PBC, we propose that determination of anti-KLHL12 antibodies can be important in the diagnostic process of PBC.

## Figures and Tables

**Table 1:** Demographic, biochemical, immunological, and histological characteristics of PBC patients and control groups.

	Primary Biliary Cholangitis Patients (*n* = 138)	Autoimmune Hepatitis Patients (*n* = 20)	Primary Sclerosing Cholangitis Patients (*n* = 40)	Healthy Adult Blood Donors (*n* = 30)
Age, years (range)	50 (26–70)	47 (19–67)	47 (23–67)	33 (19–53)
Females/males	131/7	15/5	16/24	22/8
Bilirubin (total), mg/dL	2.4 (2.2)	2.3 (2.1)	1.4 (2.6)	0.7 (0.6)
AST, U/L	81.4 (51.2)	44.3 (71.0)	97.4 (70.1)	22.5 (21.6)
ALT, U/L	93.6 (72.5)	61.7 (52.8)	86.9 (66.0)	15.1 (26.2)
AP, U/L	506.5 (429.2)	223.3 (175.4)	345.5 (227.6)	38.7 (16.8)
γ-GT, U/L	335.5 (304.2)	231.9 (204.0)	349.2 (252.4)	18.6 (4.8)
Albumin (g/dL)	3.6 (1.2)	3.4 (2.4)	2.9 (1.1)	4.5 (2.3)
γ-globulin (g/dL)	1.8 (1.1)	1.7 (1.6)	1.5 (1.8)	1.1 (0.2)
AMA M2	113 (82%)	0 (0%)	0 (0%)	0 (0%)
Anti-gp210 antibody	65 (47%)	0 (0%)	1 (2.5%)	0 (0%)
Anti-p62 antibody	39 (28%)	1 (5%)	1 (2.5%)	0 (0%)
Anti-LBR antibody	21 (15%)	0 (0%)	0 (0%)	0 (0%)
Anti-KLHL12 antibodies	49 (36%)	1 (5%)	0 (0%)	0 (0%)
Early histological stage (I/II)	82 (59%)	6 (30%)	11 (28%)	0 (0%)
Advanced histological stage (III/IV)	52 (37%)	3 (15%)	5 (13%)	0 (0%)
Ambiguous histological stage	4 (4%)	0 (0%)	0 (0%)	0 (0%)

Data are presented as mean (± SD). Abbreviations: γ-GT, γ-glutamyl transpeptidase; ALT, alanine aminotransferase; AP, alkaline phosphatase; AST, aspartate aminotransferase; Normal value: bilirubin < 1.2 mg/dL; AST < 40 U/L; ALT < 40 U/L; AP < 115 U/mg/dL; γ-GT < 50 U/L; albumin 3.5–5.5 g/dL, γ-globulin < 3 g/dL. Conversion factors to SI units are as follows: bilirubin, 17.1; AST, ALT, AP, and γ-GT, 0.0167.

**Table 2:** Diagnostic accuracy for anti-p62 and anti-gp210 antibodies in PBC patients.

	Anti-gp210	Anti-p62	Anti-LBR	Anti-NE
Sensitivity [%, 95% CI]	47.1 [38.6–55.8]	28.3 [20.9–36.6]	15.2 [9.7–22.3]	55.1 [46.4–63.5]
Specificity [%, 95% CI]	98.9 [93.9–99.9]	97.8 [92.2–99.7]	100.0 [96.0–100.0]	96.7 [90.6–99.3]
PPV [%, 95% CI]	98.5 [90.1–98.9]	95.1 [82.8–98.8]	100.0	96.2 [89.2–98.7]
NPV [%, 95% CI]	54.9 [51.0–58.8]	47.1 [44.4–49.8]	43.5 [41.8–45.2]	58.4 [53.8–62.9]
Positive Likelihood Ratio (LR+)	42.4 [6.0–300.1]	12.7 [3.2–51.4]	ND	16.5 [5.4–50.8]
Negative Likelihood Ratio (LR−)	0.5 [0.4–0.6]	0.7 [0.6–0.8]	0.9 [0.8–0.9]	0.5 [0.4–0.6]
Disease prevalence [%, 95% CI]	60.5 [53.4–65.9]	60.5 [53.9–65.9]	60.5 [53.9–66.9]	60.5 [53.9–66.9]
Accuracy [%, 95% CI]	67.5 [61.1–73.4]	55.7 [49.0–62.3]	48.7 [42.0–55.4]	71.5 [65.1–77.3]

ND—not defined.

**Table 3:** Occurrence of anti-KLHL12 antibodies in patients with PBC and control groups.

	Number of Patients	Anti-KLHL12 Antibodies
PBC	138	49 (36%)
Controls (total)	90	1 (1.1%)
PSC	40	0 (0%)
AIH	20	1 (5%)
Healthy	30	0 (0%)

**Table 4:** Data from biochemical analyses of 138 PBC patients, and the anti-NE and anti-KLHL12 antibodies status.

	Anti-NE Antibodies			Anti-KLHL12 Antibodies		
	Positive *n* = 25	Negative *n* = 68	*p*-Value	Positive *n* = 37	Negative *n* = 56	*p*-Value
Bilirubin (total), mg/dL	2.4 (2.2)	1.7 (1.6)	0.016	2.6 (2.6)	1.5 (1.1)	0.037
AST, U/L	108.8 (95.0)	72.5 (35.0)	0.008	86.9 (55.4)	68.8 (52.8)	ns
ALT, U/L	98.0 (95.4)	88.1 (75.1)	ns	95.3 (94.5)	89.1 (69.8)	ns
AP, U/L	586.7 (530.9)	398.9 (304.1)	0.036	557.3 (466.6)	429.8 (362.1)	ns
γ-GT, U/L	367.2 (345.8)	329.9 (327.7)	ns	343.4 (317.5)	310.8 (291.0)	ns

Data are presented as mean (± SD). Abbreviations: γ-GT, γ-glutamyl transpeptidase; ALT, alanine aminotransferase; AP, alkaline phosphatase; AST, aspartate aminotransferase; Normal value: bilirubin < 1.2 mg/dL; AST < 40 U/L; ALT < 40 U/L; AP < 115 U/mg/dL; γ-GT < 50 U/L; albumin 3.5–5.5 g/dL, γ-globulin < 3 g/dL. Conversion factors to SI units are as follows: bilirubin, 17.1; AST, ALT, AP, and γ-GT, 0.0167.

## Data Availability

All available data are presented within the article or are available on request from the corresponding author.

## Abbreviations

AMA: antimitochondrial antibody; AIH: autoimmune hepatitis; ALT: aspartate aminotransferase; anti-p62: anti-nucleoporin p62 antibodies ANA: antinuclear antibody, AP: alkaline phosphatase; AST: alanine aminotransferase; AUC: area under the ROC curve; EASL: European Liver Research Association; γ-GT: γ-glutamyl transpeptidase; HBsAg: hepatitis B surface antigen; IQR: interquartile range; LR−: negative likelihood ratio; NPV: negative predictive value; NE: nuclear envelope, OD: optical density; LR+: positive likelihood ratio; PPV: positive predictive value PBC: primary biliary cholangitis; PSC: primary sclerosing cholangitis; ROC: receiver operating characteristic; SD: standard deviation.
